# Circadian rhythm and cancer-related pain: a systematic review of preclinical and clinical evidence

**DOI:** 10.3389/fpain.2026.1875251

**Published:** 2026-10-26

**Authors:** Vivian Salama, Anisha Karunananthan, Joshua Pazin, Brandon M. Godinich, Michael O’Connell, Lei Wang, John C. Knoth, Ivan Martinez, Scott A. Weed

**Affiliations:** 1 Department of Medical Oncology, West Virginia University School of Medicine, West Virginia University Cancer Institute, West Virginia University, Morgantown, WV, United States; 2 Department of Radiation Oncology, West Virginia University School of Medicine, West Virginia University Cancer Institute, West Virginia University, Morgantown, WV, United States; 3 Department of Internal Medicine, Texas Health Presbyterian Hospital Dallas, Dallas, TX, United States; 4 Department of Microbiology, Immunology & Cell Biology, West Virginia University School of Medicine, West Virginia University Cancer Institute, West Virginia University, Morgantown, WV, United States; 5 Department of Biochemistry and Molecular Medicine, West Virginia University School of Medicine, West Virginia University Cancer Institute, West Virginia University, Morgantown, WV, United States

**Keywords:** cancer pain, chronotherapy, circadian rhythm, clock gene, neuroinflammation, neuropathy, opioids

## Abstract

**Background:**

Cancer-related pain and neuropathy are common complications that substantially affect quality of life. Circadian rhythms regulate biological processes involved in pain and treatment response; however, evidence linking circadian biology and treatment timing with cancer-related pain and neuropathy remains limited and heterogeneous, and the underlying mechanisms are incompletely understood. This systematic review aimed to evaluate these relationships and their potential biological mechanisms and clinical implications across clinical and preclinical studies.

**Methods:**

PRISMA-guided systematic review was registered in PROSPERO (CRD420251174524). PubMed and Web of Science were searched for original human and preclinical studies published up to October 20, 2025. Eligible studies evaluated circadian rhythms, clock genes, or circadian-based treatment timing in relation to cancer- or treatment-related pain, neuropathy, or analgesic response. Direct pain-related and indirect neuropathy outcomes were synthesized separately. Risk of bias was assessed using JBI tools for human studies and SYRCLE for preclinical studies.

**Results:**

Thirty-nine studies were included: 35 human (89.7%) and 4 preclinical studies (10.3%). Most populations included mixed cancer types (10/39, 25.6%). Cohort or observational studies represented 38.5% and clinical trials represented 28.2%, with 16/39 (41%) studies published between 2015 and 2025. Clinical findings were heterogeneous, with temporal variation reported for cancer-related pain and analgesic requirements, while chronotherapy studies suggested potential reductions in treatment-related neuropathy and neurotoxicity, representing indirect pain-related evidence. Potential mechanisms were reported or discussed in 15/35 (42.9%) human studies and were largely hypothesis-generating, involving HPA-axis/cortisol dysregulation, drug pharmacokinetics, and metabolism, inflammatory signaling, and circadian-regulatory pathways; direct clinical clock-gene evidence was limited to *RORA*. Four preclinical studies provided experimental evidence implicating clock genes (*PER2*, *BMAL1*, and *REV-ERBα/β*), oxidative stress, neuronal injury, drug metabolism, and neuroinflammatory (IL-6/LCN2) pathways. Methodological quality was limited, with high risk of bias in 10/15 (67%) cohort studies and 8/11 (73%) clinical trials and high or unclear risk across several preclinical domains.

**Conclusion:**

Current evidence suggests a potential relationship between circadian biology and cancer- and treatment-related pain, analgesic response, and neuropathy. Clinical findings remain heterogeneous, human mechanistic evidence is predominantly hypothesis-generating, and experimental evidence remains limited. Prospective mechanistic studies are needed to support future circadian-informed approaches for cancer pain management.

**Systematic Review Registration:**

https://www.crd.york.ac.uk/PROSPERO/view/CRD420251174524, identifier CRD420251174524.

## 1 Introduction

Pain is one of the most prevalent symptoms experienced by patients with cancer that can impair both physical and psychological functioning, leading to reduced quality of life (QoL) (1, 2). Pain affects up to 50% of oncology patients receiving cancer therapy, with about 90% of patients with advanced disease requiring opioid therapy (3). Pain may also coexist with fatigue, sleep disturbance, and functional decline, further increasing symptom burden (3). It may arise from the primary tumor itself, metastatic disease, or cancer treatments, including surgery, chemotherapy, and radiation therapy (4–6). Cancer pain is heterogeneous and may involve inflammatory, neuropathic, nociceptive, or mixed mechanisms (7, 8). Despite advances in cancer pain management, adequate pain control remains challenging, particularly in patients with advanced disease and treatment-related neuropathy (7, 9, 10).

Circadian rhythms are endogenous, 24 h biological oscillations driven by clock genes that regulate a wide range of physiological processes, including sleep-wake cycles, hormone secretion, immune function, metabolism, neuronal excitability, inflammatory signaling and pain sensitivity (11–14). These rhythms are coordinated by core clock genes, including *CLOCK*, *BMAL1*, *PER*, *RORA*, and *CRY* (15, 16). Circadian variation in pain sensitivity has been demonstrated in clinical and experimental studies, while disruption of circadian rhythms and clock gene expression has been associated with altered pain perception and nociceptive responses (17–20). Circadian regulation of endogenous opioid activity and hormones such as melatonin and cortisol may also contribute to time-dependent differences in pain perception and analgesic response (14, 21–25). Disruption of circadian rhythms and alterations in clock gene expression have been identified in multiple pain models, where they play significant roles in pain progression and modulation (14, 26–29).

Cancer and its treatment can disrupt circadian and sleep-wake rhythms through tumor-related inflammation, treatment effects, hospitalization, psychological stress, and changes in daily activity (1, 30, 31). This contributes to impaired daytime function and increased symptom burden (32–35). These disruptions can alter internal biological rhythms and functional systems, including endocrine, immune, and pain pathways, which have been associated with greater treatment-related adverse events, higher symptom burden including pain and fatigue, and reduced QoL (36). Several interconnected pathways may link these disturbances to cancer-related pain and neuropathy, including immune and inflammatory signaling (32, 37–42), peripheral and central sensitization (14, 27), neuroinflammation (43–45), hypothalamic-pituitary-adrenal (HPA) axis and circadian hormone dysregulation (e.g., cortisol and melatonin) (46–48), tumor-nerve interactions (48–50), psychological and sleep-related factors (51–54), altered analgesic response and drug metabolism (51, 55), and clock gene variation (56, 57) (Figure 1). These pathways suggest a potentially bidirectional relationship in which circadian disruption may influence pain and neuropathic symptoms, while pain, sleep disturbance, and treatment-related symptoms may further alter circadian function.

**Figure 1:**
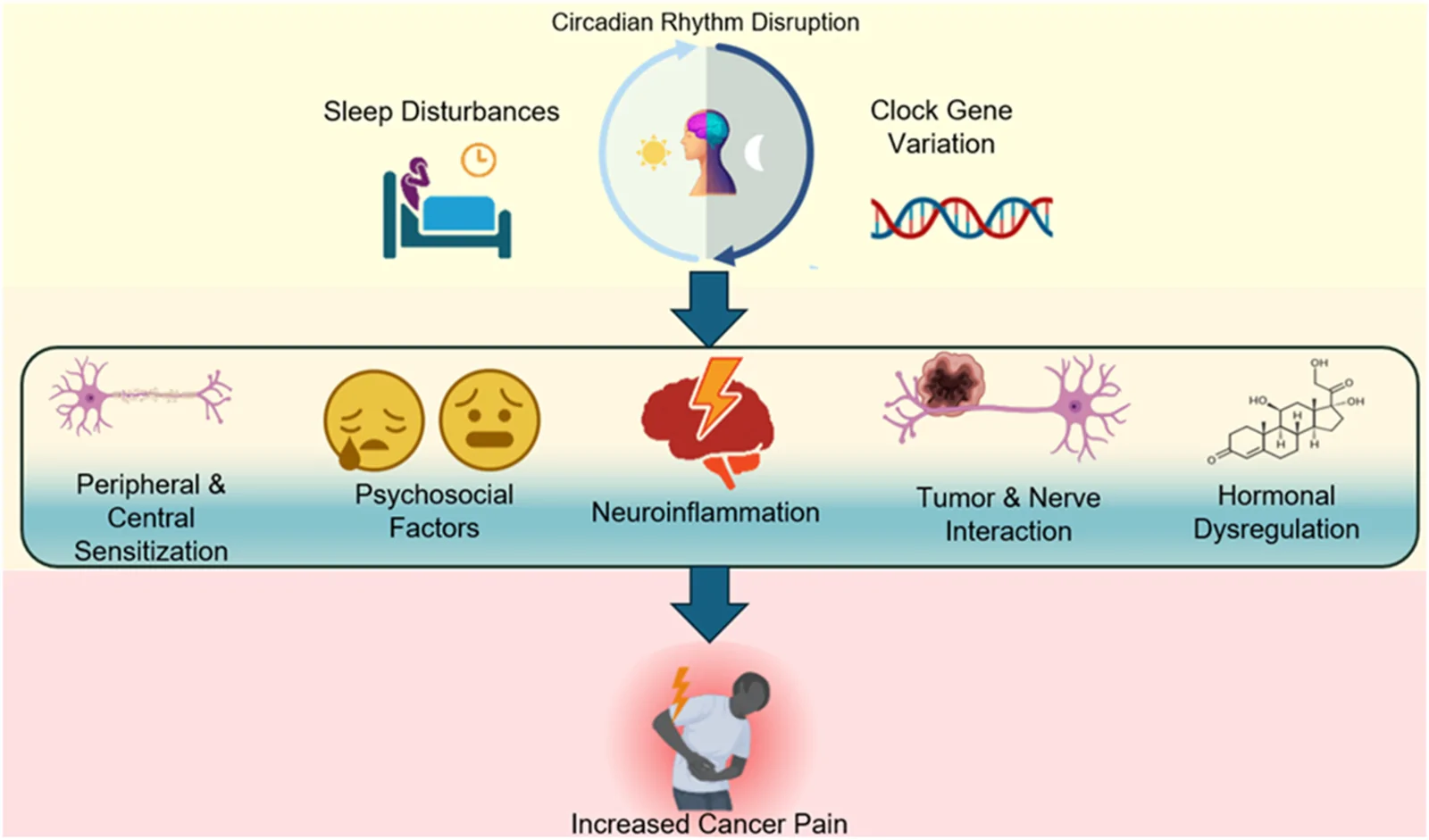
Illustration of the potential mechanisms linking circadian rhythm disruption to increased cancer pain intensity. Circadian rhythm disruption, resulting from sleep disturbances or clock gene alterations, may enhance peripheral and central nervous system sensitization, impair psychological functioning (e.g., depression and anxiety), dysregulate immune responses, and promote systemic and neuroinflammation. It may also alter the tumor microenvironment, including tumor–nerve interactions, and disrupt hormonal regulation (e.g., cortisol and melatonin secretion). These mechanisms contribute to altered pain modulation and increased cancer pain intensity.

Circadian timing may also have therapeutic implications. Chronotherapy aims to align treatment administration with biological circadian rhythms to optimize therapeutic effects or reduce toxicity (58). Clinical studies have reported time-dependent differences in treatment tolerability, neurotoxicity, pain response, and analgesic pharmacokinetics (59–63). Neuropathy is particularly relevant to cancer pain because chemotherapy-induced peripheral neuropathy is predominantly sensory and is frequently accompanied by neuropathic pain, although neuropathy may also occur without pain (64, 65). Levi et al. (62, 63) reported that chronotherapy was associated with reduced neurotoxicity and improved treatment tolerability compared with constant-rate infusion, influencing pain burden and QoL in colorectal cancer. Similarly, Chan et al. reported that radiotherapy delivered at different times of day was associated with differential pain responses in patients treated for painful bone metastases (66). Because opioids are a first-line treatment for cancer-related pain, the observed circadian variation in opioid absorption and systemic exposure may affect analgesic efficacy and adverse effect profiles (51). These findings suggest that circadian timing has clinically meaningful implications not only for therapeutic response but also for symptom burden, including pain.

together, these findings suggest that circadian biology may be relevant to cancer- and cancer treatment-related pain and neuropathy and may have implications for treatment timing and symptom management. Recent reviews have highlighted the broader relationship between circadian biology, pain, and analgesic response (67, 68). However, evidence specifically addressing circadian biology in cancer- and cancer treatment-related pain, analgesic response, and pain-relevant neuropathy remains limited and has not been comprehensively characterized across both clinical and preclinical studies. The underlying mechanisms and their potential implications for cancer pain management also remain incompletely defined.

This systematic review addressed the following questions:

1. What is the relationship between circadian rhythm disruption and cancer-related pain, neuropathy, and nociceptive or sensory outcomes in patients with cancer and preclinical cancer models?
2. What biological mechanisms have been investigated or proposed to link circadian rhythm and clock gene alterations with cancer-related pain, neuropathy, and response to analgesics?
3. What is the methodological quality and risk of bias of the available clinical and preclinical evidence?

This systematic review aimed to evaluate the relationship between circadian rhythm and cancer- and cancer treatment-related pain and neuropathy, with particular emphasis on potential biological mechanisms and clinical implications, including evidence related to treatment timing and chronotherapy identified within the included literature. We further examined the characteristics and methodological quality of the available clinical and preclinical evidence.

## 2 Materials and methods

### 2.1 Study design and protocol registration

This systematic review was conducted in accordance with the Preferred Reporting Items for Systematic Reviews and Meta-Analyses (PRISMA) guidelines. The review protocol was registered in the International Prospective Register of Systematic Reviews (PROSPERO) [ID: CRD420251174524] category for animal studies relevant to human health care and includes both human and preclinical evidence.

### 2.2 Eligibility criteria

*Inclusion criteria included:* a) full original peer-reviewed research; b) studies available in English; c) studies evaluating circadian rhythm, clock genes (as components of circadian biology), or circadian-based treatment timing (i.e., chronotherapy) in relation to cancer- or cancer treatment-related pain, neuropathy or sensory outcomes, analgesic requirements or effectiveness, or other clinically relevant pain-related symptoms/outcomes.

*Exclusion criteria included:* a) studies not related to circadian rhythm, clock gene expression/variation, or circadian-based treatment timing; b) studies evaluating pain, neuropathy, nociception, or sensory outcomes without a cancer or cancer treatment-related context; c) studies evaluating circadian rhythm, clock genes, or chronotherapy without direct pain-related outcomes or indirect neuropathy/neurotoxicity outcomes relevant to pain; d) non-original study (e.g., review article, letter, or conference abstract); e) duplicate publication, corrections or reports of the same article; and f) studies not available in English.

### 2.3 Literature search and search strategy

A comprehensive systematic search of PubMed and Web of Science databases was conducted for original publications in English up to 20th October 2025. The search combined terms related to three major concepts: (1) circadian biology, including “circadian rhythm,” and “clock gene”; (2) cancer and cancer treatment, including “cancer,” “oncology,” “neoplasm,” “tumor,” “chemotherapy,” “radiation therapy,” and “immunotherapy”; and (3) pain-related outcomes, including “pain,” “neuropathy,” “nociception,” “pain modulation,” “pain management,” “analgesics,” and “opioids.” Search terms were combined using Boolean operators (AND/OR). The complete database-specific search strategies are provided in Supplementary Materials. Additional records were identified through manual searching of Google, organizational websites, and the reference lists of included articles and relevant reviews.

### 2.4 Study selection and screening

Records identified through the database searches were imported into the Rayyan systematic review management platform, which was used to remove duplicates before screening. Two reviewers (AK and MO) independently screened titles and abstracts using a blinded screening process, with disagreements resolved by a third reviewer (VS). Potentially eligible studies subsequently underwent full-text assessment against the predefined eligibility criteria. Full-text eligibility was independently assessed by AK, MO, and VS, and disagreements were resolved through discussion and consensus.

### 2.5 Outcomes

The primary outcome of this systematic review was the relationship between circadian rhythm and cancer- or cancer treatment-related pain, neuropathy, or sensory outcomes. Secondary outcomes included associations between circadian treatment timing (chronotherapy) and pain intensity, neuropathy and analgesic requirements or response.

Pain-related outcomes were further categorized as direct or indirect according to the outcomes measured in the original studies. Direct pain-related outcomes included pain intensity or severity, breakthrough pain, painful neuropathy, allodynia or hypoalgesia, nociceptive or sensory pain outcomes, and analgesic requirements or response. Indirect pain-related outcomes included peripheral or sensory neuropathy or neurotoxicity but without direct measurement of pain.

### 2.6 Data extraction, and collection

Data from eligible studies were extracted using standardized Excel spreadsheets. Extracted variables included study demographics (author, year, and journal), study type, cancer type, subject type (human vs. non-human), population/model characteristics, sample size, study objectives, circadian or clock-related exposure/intervention, pain- or neuropathy-related outcomes, and key findings.

### 2.7 Methodological quality and risk of bias assessment

To evaluate the methodological quality of the included studies, a structured assessment of risk-of-bias was conducted using study design specific critical appraisal tools. Human studies were appraised using the critical appraisal checklist matched to their design. Cohort and other longitudinal observational studies (*n* = 15) were appraised with the JBI Checklist for Cohort Studies, clinical trials (*n* = 11) with the JBI Checklist for Randomized Controlled Trials, analytical cross-sectional studies (*n* = 6) with the revised JBI Checklist for Analytical Cross-Sectional Studies, the single qualitative focus-group study with the JBI Checklist for Qualitative Research, and the single case series with the JBI Checklist for Case Series (69–72). Design was determined from the methods reported in each article rather than from the authors’ own design label, and where the two differed the reported methods took precedence. Each checklist includes a series of signaling questions addressing key methodological domains. Each item was rated as yes, no, or unclear. Studies were classified as low risk of bias when all major methodological domains were rated “yes,” and no critical sources of bias were identified. Studies were considered moderate risk-of-bias when one or two domains were rated as “unclear” or indicated minor methodological concerns that were unlikely to substantially influence study findings. Studies were categorized as high risk-of-bias when two or more critical domains were rated as “no,” indicating a significant risk of systematic bias that could meaningfully affect the validity of the results. The single genome-wide association study (GWAS) was appraised separately using the Q-Genie tool, which covers threats specific to that design including genotyping quality, batch effects, phenotype definition, population stratification, and multiple testing (73). Q-Genie scores each of 11 items from 1 to 7 and sums them to a total between 11 and 77. A total of 35 or below indicates poor quality, above 35–45 are moderate, and above 45 are good quality (69–74).

For preclinical animal studies, risk-of-bias was assessed using the Systematic Review Centre for Laboratory animal Experimentation (SYRCLE) Risk-of-Bias Tool, which is adapted from the Cochrane risk-of-bias framework for experimental animal research (75). The SYRCLE tool evaluates domains including sequence generation, allocation concealment, baseline characteristics, random housing, blinding of investigators and outcome assessors, incomplete outcome data, selective outcome reporting, and other potential sources of bias. Each signaling question was answered Yes (low risk), No (high risk), or Unclear where insufficient detail was reported to judge the item, with Not applicable used where a question did not apply to the design. Each of the ten domains then received a judgement of low, high, or unclear risk.

### 2.8 Data and evidence synthesis

Given the heterogeneity in included study designs, populations, outcome measures, and methodologies, a quantitative meta-analysis was not feasible. Therefore, a qualitative (narrative) synthesis approach was employed. All included studies were categorized into two main groups based on the subject type: (1) studies conducted on human subjects (clinical studies), (2) studies conducted on non-human subjects (preclinical studies). Evidence was further synthesized according to outcome type, circadian or clock-related factors, chronotherapy and proposed mechanistic pathways to identify consistent findings, areas of heterogeneity, and gaps in literature. Direct and indirect pain-related evidence was interpreted separately.

### 2.9 Results reporting and presentation

Results were summarized in tabular and narrative format to highlight study characteristics, methodological features and reported key findings. Descriptive visualizations (e.g., bar charts, pie charts, and flow diagrams) were generated using GraphPad Prism version 10.5.0 and Microsoft Excel to illustrate the study distribution, cancer types, and type of research study. Traffic light plots were used for visualization of Risk of bias assessment using Roves visualization tool (https://www.riskofbias.info/welcome/robvis-visualization-tool).

## 3 Results

### 3.1 Search and screening results

The database search identified 190 records, comprising 108 from PubMed and 82 from Web of Science. Ten additional records were identified through other sources (Google search, *n* = 2; organizational websites, *n* = 1; reference lists of reviews, *n* = 7). Thirty-nine articles met all inclusion criteria. The full search process is illustrated in the PRISMA flow chart (Figure 2).

**Figure 2:**
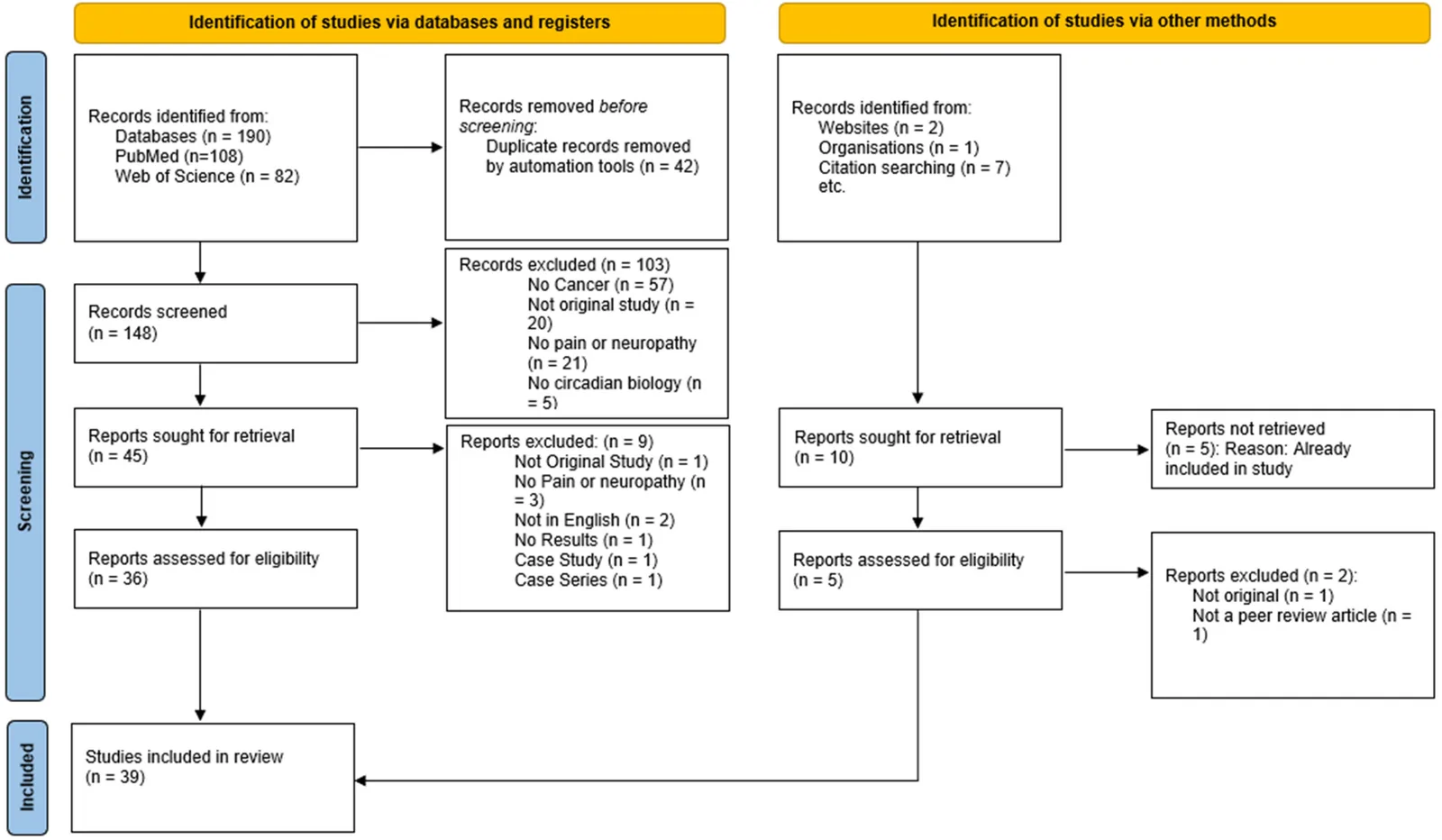
PRISMA flow chart of systematic review of circadian rhythm, clock genes and cancer pain.

### 3.2 Studies characteristics

The most frequently represented cancer categories were mixed cancer types (10/39, 25.6%) and advanced cancer (9/39, 23.1%), followed by colorectal cancer (8/39, 20.5%) and breast (6/39, 15.4%) (Figure 3A). The included studies used a range of designs (Figure 3B). Preclinical studies were used in 4/39 (10.3%). Among the 35 human studies, 15 were cohort or other longitudinal observational designs, 11 were clinical trials, 6 were analytical cross-sectional studies, and one each was a case series, a qualitative focus-group study, and a genome-wide association study. Four preclinical studies (4/39, 10.3%) were included. Figure 3.C illustrates the publication trend of included studies in this review. The earliest article was published in 1988 and the latest analyzed publication in 2025. Demographics, characteristics, conclusions, and potential mechanisms are summarized in Table 1.

**Figure 3:**
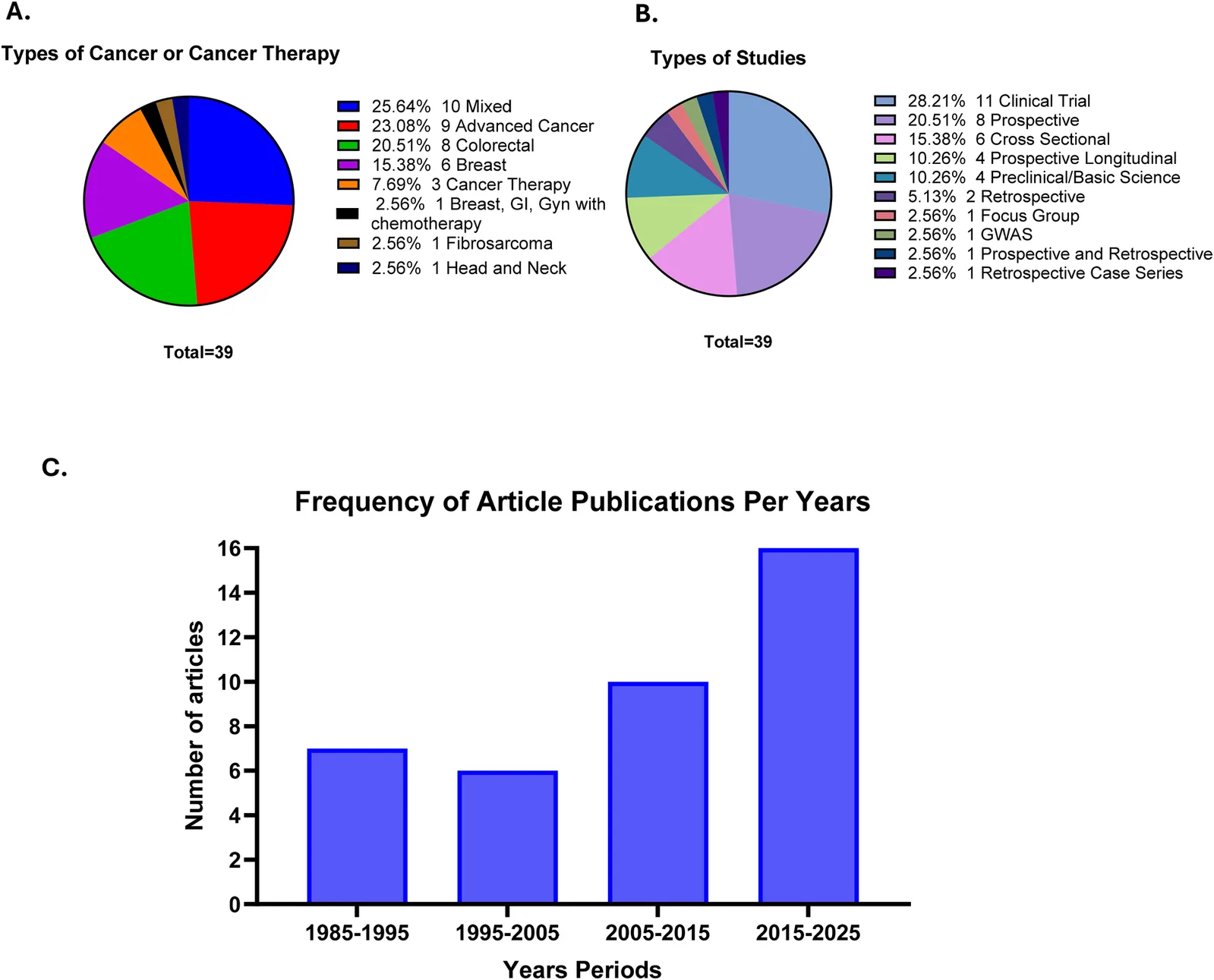
Publication trend and characteristics of studies included in this review. **(A)** Frequency of types of cancer or cancer therapy included in the studies included in this review. **(B)** Frequency of types of studies in the included studies. **(C)** Trend of the publications per 10-year periods.

**Table 1:** Demographics and characteristics of included studies in this systematic review.

Author, year	Journal	Cancer or cancer treatment type	Type of study	Participants/models	Conclusion/results	Potential mechanism
Clinical/human studies						
Barni et al. (76)	Tumori	Mixed	Cross-Sectional	163—(121 cancer, 42 healthy control)	Circadian rhythm was altered in cancer patients for opioid but not B-endorphin	HPA axis dysregulation
Levi et al. (60)	Cancer	Colorectal	Clinical Trial	93	Chronotherapy reduced toxicities (peripheral neuropathy)	Not Reported
Bruera et al. (77)	Pain	Advanced Cancer	Prospective and Retrospective	61	Cancer patients require more narcotics during daytime	Not Reported
Citron et al. (78)	Cancer Investigations	Advanced Cancer	Prospective	16	Lower analgesic demand during nighttime	Not Reported
Levi et al. (62)	European Journal of Cancer	Colorectal	Clinical Trial	29	Chronotherapy allowed higher dose intensity, manageable toxicity (peripheral neuropathy)	Not Reported
Levi et al. (59)	Journal of the National Cancer Institute	Colorectal	Clinical Trial	92	Chrono modulated therapy allowed less severe toxicities compared to fixed-rate infusion (peripheral neuropathy)	Not Reported
Gourlay et al. (51)	Pain	Mixed	Clinical Trial	26	Plasma morphine concentration varies significantly throughout the day	Changes in absorption from the upper regions GIT or changes in the volume of distribution for morphine
Levi et al. (63)	Lancet	Colorectal	Clinical Trial	186	Chronotherapy decreased toxicities (peripheral neuropathy) compared with constant-rate infusion	Drug metabolism
Levi et al. (79)	Cancer	Colorectal	Clinical Trial	90	Chronotherapy brought a two-fold improvement in neurologic tolerability	DNA synthesis and drug metabolizing enzyme activity
Klepstad et al. (80)	Methods and Findings in Experimental and Clinical Pharmacology	Mixed	Prospective	40	No significant circadian rhythm variation of opioids	Not Reported
Giacchetti et al. (81)	Journal of Clinical Oncology	Colorectal	Clinical Trial	200	Adding oxaliplatin to chronomodulated fluorouracil–leucovorin as first-line treatment reduced peripheral neuropathy when compared to continuous flat infusion or 2 h flat infusion	Not Reported
Gagnon et al. (82)	Journal of Pain and Symptom Management	Advanced Cancer	Prospective	104	Breakthrough analgesia (BTA) use follows a circadian pattern higher during the day in non-delirious patients but shifts toward evening and nighttime predominance in patients with delirium	Not Reported
Curran et al. (83)	Journal of Behavioral Medicine	Breast	Prospective	25 (25 breast cancer, 25 healthy controls and 24 benign breast problem controls)	No cancer-specific alteration in circadian rhythm of pain	Not Reported
Gholam et al. (84)	Oncologist	Colorectal	Clinical Trial	77	chronomodulated ambulatory chemotherapy in causing peripheral neuropathy was inconclusive due to previous baseline sensory neuropathy scores from previous oxiplatin treatment	DNA synth, cell-cycle regulation, and metabolism
Currow et al. (85)	Journal of Pain and Symptom Management	Mixed	Clinical Trial	42 (26 complete, 16 withdrew)	No significant difference in pain control between morning or evening morphine	Steady-state sustained-release dosing likely buffers these chronobiological differences, minimizing clinical impact
Bouchahda et al. (86)	Cancer	Colorectal	Retrospective	29	Chronomodulated oxaliplatin-related sensory neuropathy was aggravated in only 13.8% of patients but were stable in the majority of patients or even improved in 20.6%	Higher hepatic clearance (metabolism) of oxaliplatin administered by HAI administration versus iv administration
Dhruva et al. (87)	Cancer Nursing	Breast	Prospective Longitudinal	73	Pain was a potential predictor for morning fatigue in breast cancer patients	Not Reported
Flynn et al. (34)	Psycho-Oncology	Mixed	Focus group	67	Sleep was affected by pain, with some experiencing burns/scars or muscle/joint/bone pain inhibiting sleep, and others taking pain medications for pain causing oversleeping	Not Reported
Buffum et al. (88)	The Journal of Pain	Mixed	Cross-Sectional	182	Males in pain reported significantly higher daytime dysfunction scores than males without pain. The pain group had larger proportion of breast cancer patients than the no pain group which had more prostate cancer patients	Not Reported
Rosen et al. (89)	Supportive Care in Cancer	Mixed	Retrospective case series	70 (children)	In several children, pain was the primary cause of insomnia and improved when adequate pain control was provided.	Not Reported
Saini et al. (30)	European Journal of Pain	Advanced Cancer	Prospective	123	breakthrough pain (BTP) demonstrated a significant and reproducible circadian rhythm with a late morning acrophase correlating with worse QoL	Not Reported
Ma et al. (1)	Supportive Care in Cancer	Advanced Cancer	Cross-Sectional	68	Dampened circadian rest–activity rhythms were significantly associated with higher pain intensity and poorer sleep quality, with pain functioning as a complete mediator between circadian disruption and sleep disturbance	HPA-axis dysregulation
Chang et al. (90)	Chronobiology International	Advanced Cancer	Prospective	68	Disruptions in rest activity rhythm were negatively correlated with the survival of hospitalized cancer patients experiencing pain, particularly among patients with poor performance status	HPA-axis dysregulation
Abid et al. (33)	Journal of Pain and Symptom Management	Mixed	Prospective longitudinal	1,333	Pain was not statistically significant in accounting for lower sleep disturbances, lower functional status, and poorer attentional function	Not Reported
Chan et al. (66)	Annals of Palliative Medicine	Mixed	Retrospective	194	Time of treatment led to significantly improved response rates in females with painful bone metastases. However, this was not observed in male participants or in all participants combined	Circadian rhythms regulate bone remodeling (osteoblast/osteoclast activity), DNA synthesis, and cell-cycle protein expression. Sex-specific differences in circadian sensitivity may alter tumor radiosensitivity
Sultan et al. (91)	Chronobiology International	Breast	Cross-Sectional	25	Progressive chemotherapy exposure was associated with significant worsening of circadian rest–activity rhythm and increased pain	Not Reported
Reyes-Gibby et al. (56)	Nature—Scientific Reports	Head and Neck	Genome-Wide Association Study	1,043	*RORA* is 1 of 4 genes associated with neuropathy in head and neck cancer patients	Expression of *RORA* in lamina II of dorsal horn of the spinal cord, a region for somatosensory signals of touch, temperature, and pain
Ho et al. (92)	Psychoneuroendocrinology	Breast	Clinical Trial	121 (63 DMT, 58 control)	Pain severity was not significantly associated with circadian rhythm	Not Reported
Bernatchez et al. (35)	Chronobiology International	Advanced Cancer	Cross-Sectional	55	Circadian rhythm patterns did not demonstrate a statistically significant association with pain	Not Reported
Campagna et al. (31)	Cancers	Advanced Cancer	Prospective Longitudinal	92	Breakthrough pain episodes demonstrated a significant circadian rhythm with a late-morning acrophase independent of care setting, metastasis site, or performance status	Not Reported
Knoerl et al. (93)	Oncology Nursing Forum	Breast, GI, Gyn with chemo induced peripheral neuropathy	Cross-Sectional	13	Individuals with chronic chemotherapy-induced peripheral neuropathy demonstrated a weaker cortisol awakening response and a blunted daily cortisol rhythm	HPA axis dysregulation
Scher et al. (94)	JMIR Formative Research	Mixed	Prospective	905	Sleep disorders were highly prevalent and significantly associated with pain and poorer QoL	Immuno-inflammatory system activation
Shin et al. (36)	Biological Rhythm Research	Breast	Prospective Longitudinal	27	Circadian misalignment prior to chemotherapy was significantly associated with higher global, sensory, and motor CIPN severity during and up to 9 months after treatment	Neuroinflammation through immune dysregulation
Wells-Di Gregorio et al. (95)	Psycho-Oncology	Advanced Cancer	Clinical Trial	21	6-week CBT-ACT multi-symptom intervention (FOCUS) significantly improved objective sleep efficiency, reduced wake after sleep onset, awakenings, and napping, and reduced pain by half	Not Reported
Shin et al. (96)	Chronobiology International	Breast	Prospective	23	Greater circadian misalignment was significantly associated with more severe global and sensory chemotherapy-induced peripheral neuropathy	Inflammatory responses and neuroinflammation
Preclinical/Non-Human Studies						
Kim et al. (97)	Scientific Reports	Cancer therapy	Preclinical	Paclitaxel-treated rats (8 paclitaxel-treated, 8 vehicle controls); Per2::LucSV reporter and C57BL/6J mice for *ex vivo* studies	Paclitaxel-induced neuropathic pain exhibited significant circadian variation, with the greatest pain hypersensitivity during the daytime/inactive phase. Paclitaxel also altered circadian rhythmicity in DRG neurons and produced time-of-day-dependent transcriptomic changes in DRG and dorsal horn tissues.	[Clock genes/circadian regulation; neuronal and peripheral nervous system pathways; neuroinflammation] Peripheral sensory tissues possess intrinsic circadian clocks involving *PER2* and *BMAL1*. Paclitaxel lengthened the circadian period in DRG neurons and altered the diurnal expression of core clock and clock-controlled genes. Circadian and paclitaxel-responsive transcriptional pathways substantially overlapped in the DRG and dorsal horn.
Seto et al. (98)	European Journal of Pharmacology	Cancer therapy	Preclinical	16 Sprague-Dawley rats (12 experimental, 4 control)	Repeated cisplatin administration caused time-dependent differences in peripheral neuropathy severity, increased neuronal apoptosis markers, and more severe hypoalgesia, while antioxidant treatment (DMTU) attenuated oxidative stress and prevented hypoalgesia	[Oxidative stress and neuronal injury]. Dosing-time differences alter cisplatin-induced reactive oxygen species (ROS) production, leading to mitochondrial dysfunction, neuronal apoptosis in DRG/sciatic nerve tissue, and subsequent hypoalgesia.
Seto et al. (99)	Anticancer Research	Cancer therapy	Preclinical	100 Male C57BL/6J mice (76 experimental, 24 control)	Paclitaxel-induced mechanical allodynia demonstrated significant circadian variation in producing severe/prolonged neuropathy, and repeated 21:00 administration leading to earlier recovery without worsening liver toxicity	[Time-dependent drug metabolism]. Circadian differences in paclitaxel metabolism may underlie the time-dependent severity of neuropathy, potentially mediated by diurnal variation in CYP2C and CYP3A enzyme activity
Yasukochi et al. (100)	The Journal of Neuroscience	Fibrosarcoma	Basic Science	Sample sizes ranged from *n* = 3–8 mice per group across experiments; however, the total number of animals used was not explicitly reported.	Cancer-induced neuropathic pain (tactile allodynia) shows circadian (diurnal) variation for NCTC2472 fibrosarcoma mice but not control mice	[Circadian regulation of neuroinflammatory signaling] Tumor growth elevates spinal IL-6, inducing microglial LCN2 expression that is negatively regulated by REV-ERBα/β. Oscillations in LCN2 levels contribute to diurnal variation in cancer-induced tactile allodynia, with functional validation via antibody inhibition and recombinant LCN2 administration.

### 3.3 Clinical human studies

Thirty-five human studies (89.74%) were included, encompassing heterogeneous cancer populations, study designs, circadian exposures, and pain- or neuropathy-related outcomes. The clinical evidence was broadly categorized into studies evaluating circadian patterns of pain and analgesic use, circadian disruption and pain-related symptoms, chronotherapy and treatment-related neuropathy/neurotoxicity, circadian disruption and chemotherapy-induced peripheral neuropathy (CIPN), and clock gene-related evidence. Several studies reported direct pain-related outcomes, whereas others evaluated neuropathy or neurotoxicity without direct measurement of pain; these findings were considered separately.

#### 3.3.1 Circadian rhythm and cancer-related pain

Several human studies investigated associations between circadian rhythm, rest-activity patterns, sleep, and cancer-related pain, although findings were heterogeneous. Saini et al. (30) identified a significant and reproducible circadian pattern in breakthrough cancer pain, characterized by late morning atrophies and an association with poorer QoL. Similarly, Campagna et al. (31) reported a significant circadian pattern of breakthrough pain episodes with a late morning atrophy, which was observed independently of care setting, metastatic site, or performance status.

Alterations in rest-activity rhythms were also associated with pain-related outcomes. Ma et al. (1) reported higher pain intensity among patients with dampened circadian rest-activity rhythms, while Chang et al. (90) found that disruption of rest-activity rhythms was negatively associated with survival among hospitalized patients with advanced cancer experiencing pain, particularly those with poor performance status. Sultan et al. (91) additionally reported that progressive chemotherapy exposure was associated with worsening rest-activity rhythms and increased pain in patients with breast cancer.

Other studies examined the relationship between pain and sleep or circadian disruption from the opposite direction, suggesting that pain itself may contribute to sleep or rest-activity disturbances. Scher et al. (94) reported an association between sleep disorders and pain, while Rosen et al. (89) described improvement in sleep problems following improved pain control in pediatric patients with cancer. These findings suggest a possible bidirectional interaction between pain and circadian or sleep disruption; however, the observational nature of the evidence does not establish a causal relationship.

In contrast, several studies did not identify significant associations between circadian or sleep-related measures and pain. Curran et al. (83) found no cancer-specific alteration in the circadian pattern of pain among patients with breast cancer. Ho et al. (92) reported that pain severity was not significantly associated with circadian rhythm measures in patients with breast cancer, while Bernatchez et al. (35) similarly found no statistically significant association between circadian rhythm patterns and pain in patients with advanced cancer. Abid et al. (33), reported that pain was not significantly associated with sleep disturbance in their study population. Collectively, these findings indicate that temporal patterns of cancer-related pain have been observed in some populations, particularly for breakthrough pain, but associations between broader circadian disruption and pain have not been consistently demonstrated across studies.

#### 3.3.2 Circadian variations in analgesic and opioid use

Seven human studies evaluated circadian or time-of-day variation in analgesic use, opioid requirements, or opioid-related pharmacokinetics. Bruere et al. (77) determined that patients with advanced cancer required more narcotics during the daytime, and Citron et al. (78) observed lower analgesic demands during the night. Barni et al. (76) reported altered circadian rhythms of opioid peptides, but not beta-endorphin, in patients with cancer.

#### 3.3.3 Chronotherapy and treatment-related neuropathy and neurotoxicity

Eight chronotherapy studies evaluated peripheral neuropathy or neurologic toxicity. Several trials of chronomodulated chemotherapy in colorectal cancer reported reduced peripheral neuropathy, improved neurologic tolerability, or more manageable treatment toxicity compared with conventional or constant-rate treatment schedules. Levi et al. reported reduced treatment-related toxicities, including peripheral neuropathy, with chrono-chemotherapy across several trials [Levi et al. (59, 60, 63)]. An earlier study by Lévi et al. demonstrated that chronotherapy permitted increased dose intensity with manageable toxicity [Levi et al. (62)]. Subsequently, Levi et al. (79) found a two-fold improvement in neurologic pain tolerability with chronomodulated 5-fluorouracil, leucovorin and oxaliplatin. Giacchetti et al. (81) similarly reported reduced peripheral neuropathy with chronomodulated oxaliplatin-containing therapy compared with continuous or flat infusion schedules.

Not all chronotherapy findings were consistent. Gholam et al. (84) reported inconclusive findings regarding peripheral neuropathy partly due to pre-existing sensory neuropathy from prior oxaliplatin exposure, whereas Bouchahda et al. (86) reported worsening sensory neuropathy in 13.8% of patients, stability in the majority, and improvement in 20.6%. Because these studies assessed neuropathy or neurologic toxicity without directly measuring pain, they were considered separately from direct pain-related evidence.

#### 3.3.4 Circadian disruption and peripheral neuropathy

More recent studies evaluated the relationship between circadian disruption and chemotherapy-induced peripheral neuropathy (CIPN). Knoerl et al. (93) reported that patients with chronic CIPN pain demonstrated a weaker cortisol awakening response and a blunted daily cortisol circadian rhythm. Shin et al. (36, 96) also reported associations between circadian misalignment and more severe CIPN in two studies. These observational findings demonstrate associations between altered circadian measures and CIPN severity but do not establish a causal relationship.

#### 3.3.5 Clock gene-related evidence

Direct clinical evidence involving circadian clock genes was limited. In a genome-wide association study of 1,043 patients with head and neck cancer (HNC), Reyes-Gibby et al. (56) identified *RORA*, a circadian clock-related gene, among four genes associated with neuropathy.

#### 3.3.6 Hypothesis-generating mechanisms from clinical evidence

Potential biological or pharmacological mechanisms were reported or discussed in 15 of the 35 human studies (42.9%). However, mechanistic evidence was predominantly hypothesis-generating, with most studies proposing potential pathways to explain the observed clinical associations rather than directly testing causal mechanisms. Several recurring mechanistic pathways emerged from clinical evidence.

1. HPA-axis and cortisol dysregulation was reported or proposed in four studies [Barni et al. (76), Ma et al. (1), Chang et al. (90), and Knoerl et al. (93)]. Barni et al. proposed HPA-axis dysregulation in relation to altered circadian opioid patterns, while Ma et al. and Chang et al. proposed HPA-axis disruption as a potential mechanism linking altered rest-activity rhythms with pain-related outcomes. Knoerl et al. provided physiological evidence by measuring daily salivary cortisol patterns in patients with chronic CIPN pain, demonstrating a weak cortisol awakening response and blunted daily cortisol rhythm.
2. Circadian variation in drug pharmacokinetics and metabolism was discussed in six studies [Gourlay et al. (51), Levi et al. (63), Levi et al. (79), Gholam et al. (84), Currow et al. (85), and Bouchahda et al. (86)]. Proposed mechanisms included time-dependent gastrointestinal absorption and morphine distribution, circadian variation in drug metabolism and metabolizing-enzyme activity, DNA synthesis and cell-cycle regulation, and hepatic clearance of oxaliplatin. Currow et al. additionally proposed that steady-state sustained-release morphine dosing may attenuate underlying chronobiological differences.
3. Immune-inflammatory and neuroinflammatory pathways were proposed in three studies [Scher et al. (94), Shin et al. (36), and Shin et al. (96)]. These studies suggested that immune-inflammatory activation or neuroinflammation may contribute to associations between circadian disruption and pain- or CIPN-related outcomes.
4. Clock-gene and circadian-regulatory pathways were represented by Reyes-Gibby et al. (56), who identified *RORA*, a circadian-regulatory gene, among genes associated with neuropathy in patients with HNC. The authors noted *RORA* expression in lamina II of the spinal dorsal horn, a region involved in somatosensory signals of pain, touch and temperature, provided a potential biological link between circadian regulation and sensory outcomes (101).
5. Circadian regulation of bone remodeling, DNA synthesis, and cell-cycle activity, together with potential sex-specific differences in circadian sensitivity, was proposed by Chan et al. (66) as a potential mechanism underlying time-of-day differences in pain response to radiotherapy for painful bone metastases.

Overall, these clinical findings generate several biological potential hypotheses linking circadian biology with cancer-related pain, analgesic response, and treatment-related neuropathy; however, most mechanisms were proposed to explain observed clinical associations rather than directly tested as causal pathways.

### 3.4 Preclinical non-human studies

Four preclinical studies (4/39, 10.3%) investigated circadian or dosing-time effects on cancer- or cancer treatment-related pain and neuropathy, providing experimental evidence implicating molecular clock regulation, oxidative stress, drug metabolism, neuronal injury, and neuroinflammatory pathways.

Kim et al. (97) demonstrated a circadian pattern of paclitaxel-induced peripheral neuropathic pain in a preclinical rat model. Male Sprague-Dawley rats treated with paclitaxel exhibited significant time-of-day variation in mechanical allodynia, assessed using von Frey testing, with greater pain hypersensitivity during the daytime/inactive phase. The authors evaluated molecular clock activity in dorsal root ganglion (DRG) and dorsal horn tissues using circadian reporter and transcriptomic analyses. DRG neurons exhibited intrinsic circadian oscillations involving core clock components, including *PER2* and *BMAL1*, while paclitaxel exposure altered DRG circadian rhythmicity and produced time-dependent changes in clock- and pain-related gene expression. Transcriptomic analysis further demonstrated substantial overlap between genes exhibiting diurnal rhythmicity and those altered by paclitaxel in both the DRG and dorsal horn. These findings suggest that chemotherapy-induced neuropathic pain is modulated by interactions between the molecular circadian clock and neuronal, inflammatory, and pain-signaling pathways, providing a mechanistic basis for time-of-day variation in paclitaxel-induced neuropathic pain.

Seto et al. (98) demonstrated time-of-day differences in cisplatin-induced peripheral neuropathy and hypoalgesia in rats. This study used a male Sprague-Dawley rat model of cisplatin-induced peripheral neuropathy, in which cisplatin was administered at various times during the day once weekly for four weeks to evaluate dosing-time-dependent differences in hypoalgesia and oxidative stress (98). Pain behavior was assessed using the hot-plate test, while oxidative stress in the sciatic nerve was quantified via TBARs (lipid peroxidation) and SOD activity, and apoptosis-related gene expression (p53, Bax/Bcl2) was measured in dorsal root ganglia using qRT-PCR. Repeated cisplatin administration produced dosing-time-dependent differences in neuropathy severity, neuronal apoptosis markers, and hypoalgesia. Mechanistically, differences in cisplatin-induced reactive oxygen species production were associated with mitochondrial dysfunction and neuronal apoptosis in dorsal root ganglion and sciatic nerve tissues. Oxidative stress appeared to be a key determinant of peripheral neuropathy, and the 17:00 dosing regimen was associated with higher oxidative stress, suggesting that dosing time may influence neuropathy severity.

A subsequent study by Seto et al. (99) similarly demonstrated circadian variation in paclitaxel-induced peripheral neuropathy in mice. Paclitaxel is known to damage nerve fibers. Male C57BL/6J mice received paclitaxel at different times of day, and mechanical allodynia was assessed. Mechanical sensitivity was quantified using the Von Frey up-down method to calculate the 50% withdrawal threshold, and repeated dosing regimens were used to mimic clinical exposure. Plasma paclitaxel concentrations were measured via LC-MS/MS to assess pharmacokinetic variation, alongside body weight and ALT levels to evaluate systemic toxicity, thereby allowing investigation of circadian influences on neuropathy severity and drug metabolism. Neuropathy severity varied significantly by dosing time, with administration at 9:00 producing more severe and prolonged mechanical allodynia. Chronotherapy improved recovery from neuropathy and was associated with lower paclitaxel plasma concentrations at later time points. Circadian variation in paclitaxel metabolism, potentially involving diurnal differences in CYP2C and CYP3A enzyme activity, was proposed as a mechanism underlying the time-dependent differences in neuropathy severity.

Finally, Yasukochi et al. (100) demonstrated circadian variation in cancer-induced neuropathic pain in the fibrosarcoma mouse model. Tumor-bearing mice exhibited pronounced tactile allodynia with clear diurnal variation, characterized by heightened pain sensitivity during specific phases of the light-dark cycle. Molecular analyses of spinal cord tissue revealed that tumor growth increased IL-6 levels, which in turn induced expression of lipocalin-2 (LCN2) in spinal microglia. Temporal profiling demonstrated rhythmic oscillations of LCN2 expression that were inversely correlated with the activity of circadian repressors REV-ERBα and REV-ERBβ. Functional experiments, including intrathecal administration of LCN2-neutralizing antibodies and recombinant LCN2, confirmed that LCN2 directly modulates pain sensitivity, with inhibition reducing and exogenous administration exacerbating tactile allodynia. Additional mechanistic studies using lentiviral overexpression and pharmacological activation (SR9009) of REV-ERBs demonstrated suppression of LCN2 expression and attenuation of cancer-associated pain, indicating that circadian regulation of microglial signaling contributes to diurnal fluctuations in neuropathic pain.

Collectively, these preclinical studies provide experimental evidence that circadian modulation of cancer- and treatment-related pain phenotypes may involve interactions among clock-gene and neuronal signaling, oxidative stress, time-dependent drug metabolism, and neuroinflammatory processes.

### 3.5 Risk of bias

The 35 human studies were appraised with design-matched checklists (15 cohort studies, 11 clinical trials, 6 analytical cross-sectional studies, 1 case series, 1 qualitative study, and 1 genome-wide association study). Risk of bias was high in most cohort studies and clinical trials, and confounding was the dominant weakness across the cross-sectional evidence. Among the cohort studies (*n* = 15), 10/15 (67%) were classified as high risk-of-bias, 3/15 (20%) as low risk, and 2/15 (13%) as moderate risk. The most frequent methodological limitations were concentrated in the baseline outcome-free (10/15, 67% “No”), handling missing data (10/15, 67% “No”), confounding (confounder adjustment: 10/15, 67% “No”), exposure consistency (6/15, 40% “Unclear”) and follow-up domains (4/15, 27% “Unclear”). Specifically, many studies failed to clearly identify relevant confounding variables or implement analytic strategies to address them, and several did not report whether follow-up duration was sufficient for outcomes to occur. In addition, incomplete follow-up, and lack of transparency regarding attrition were common, with many studies not reporting reasons for loss to follow-up or statistical approaches to address missing outcome data (Figure 4A).

**Figure 4:**
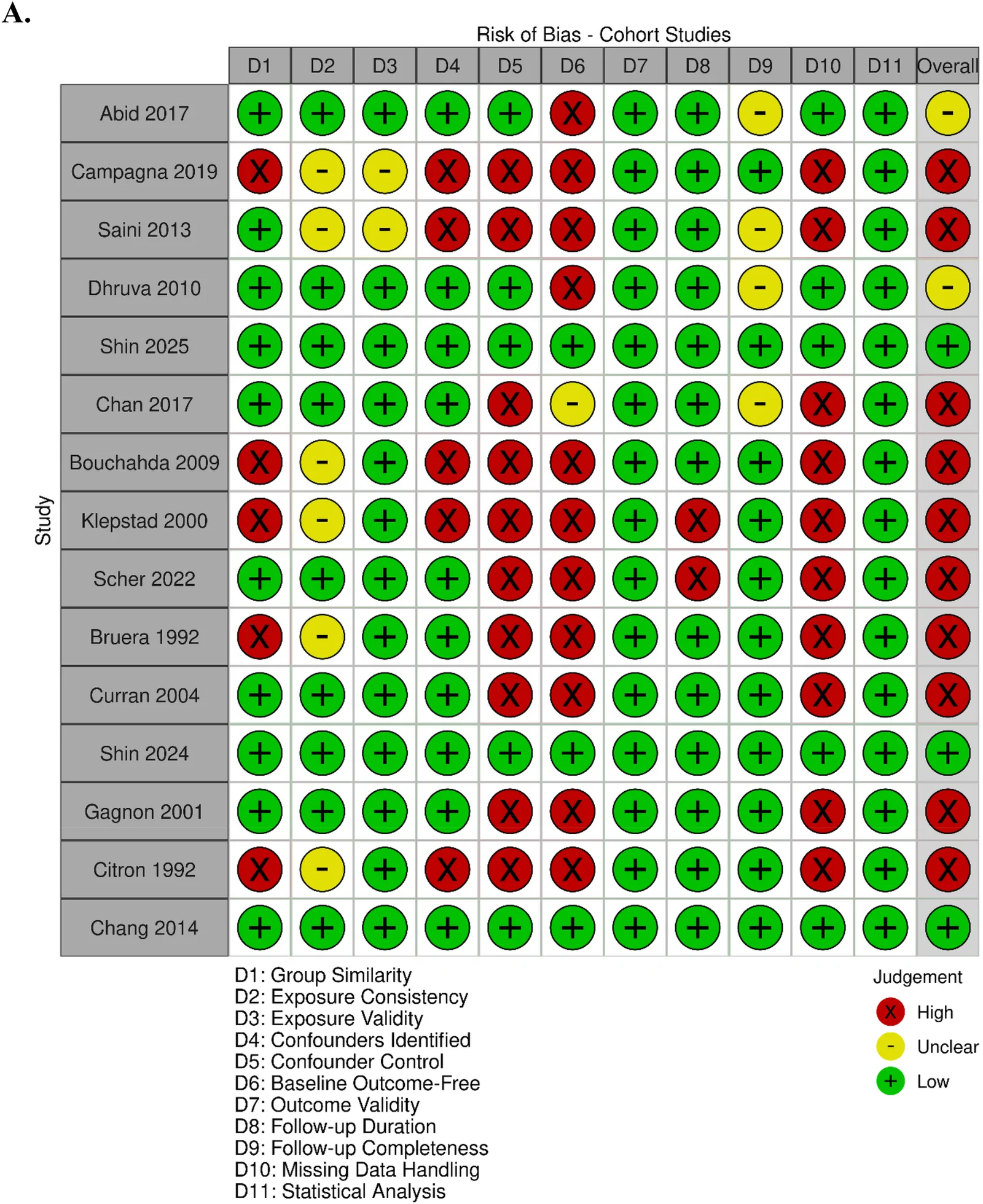

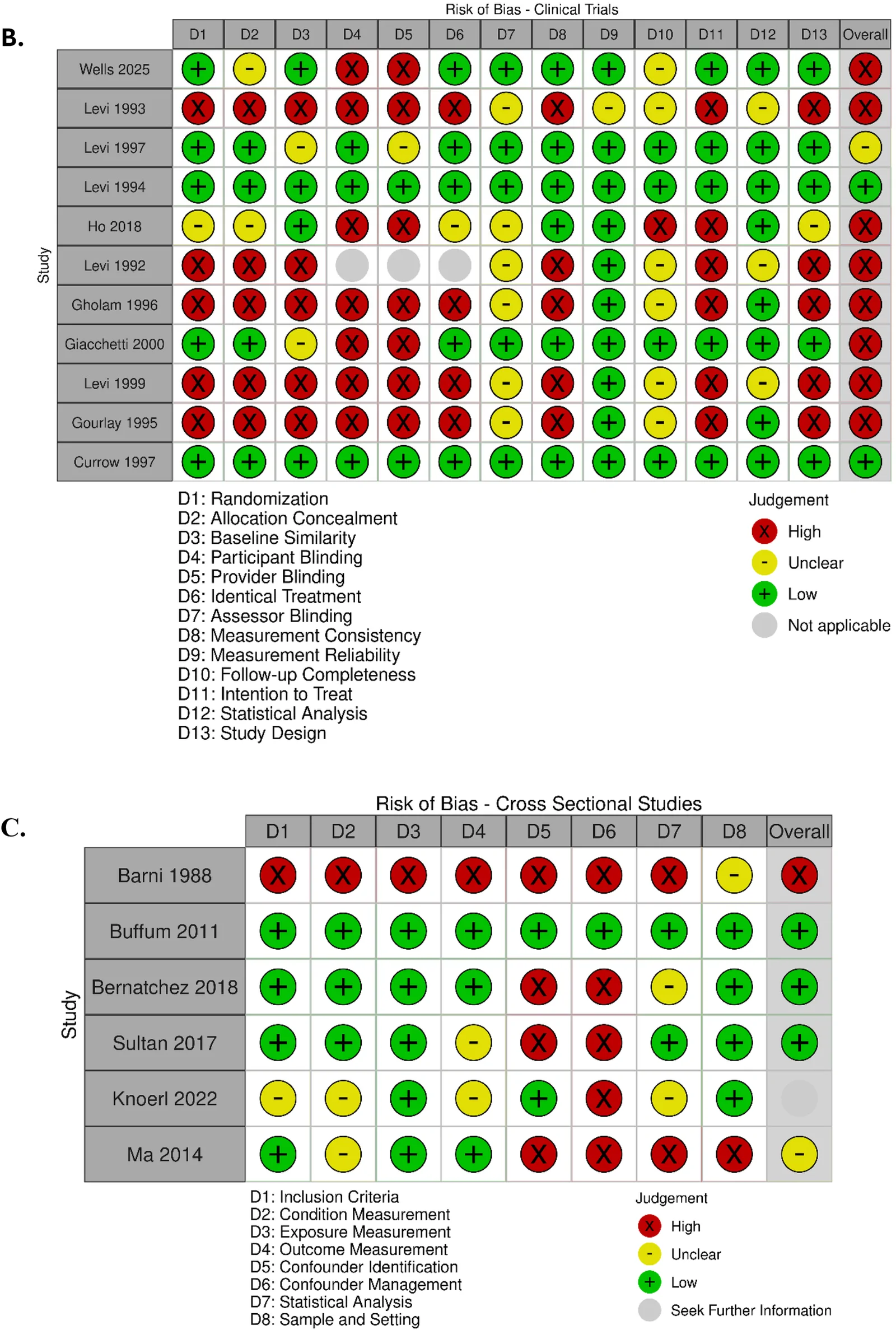

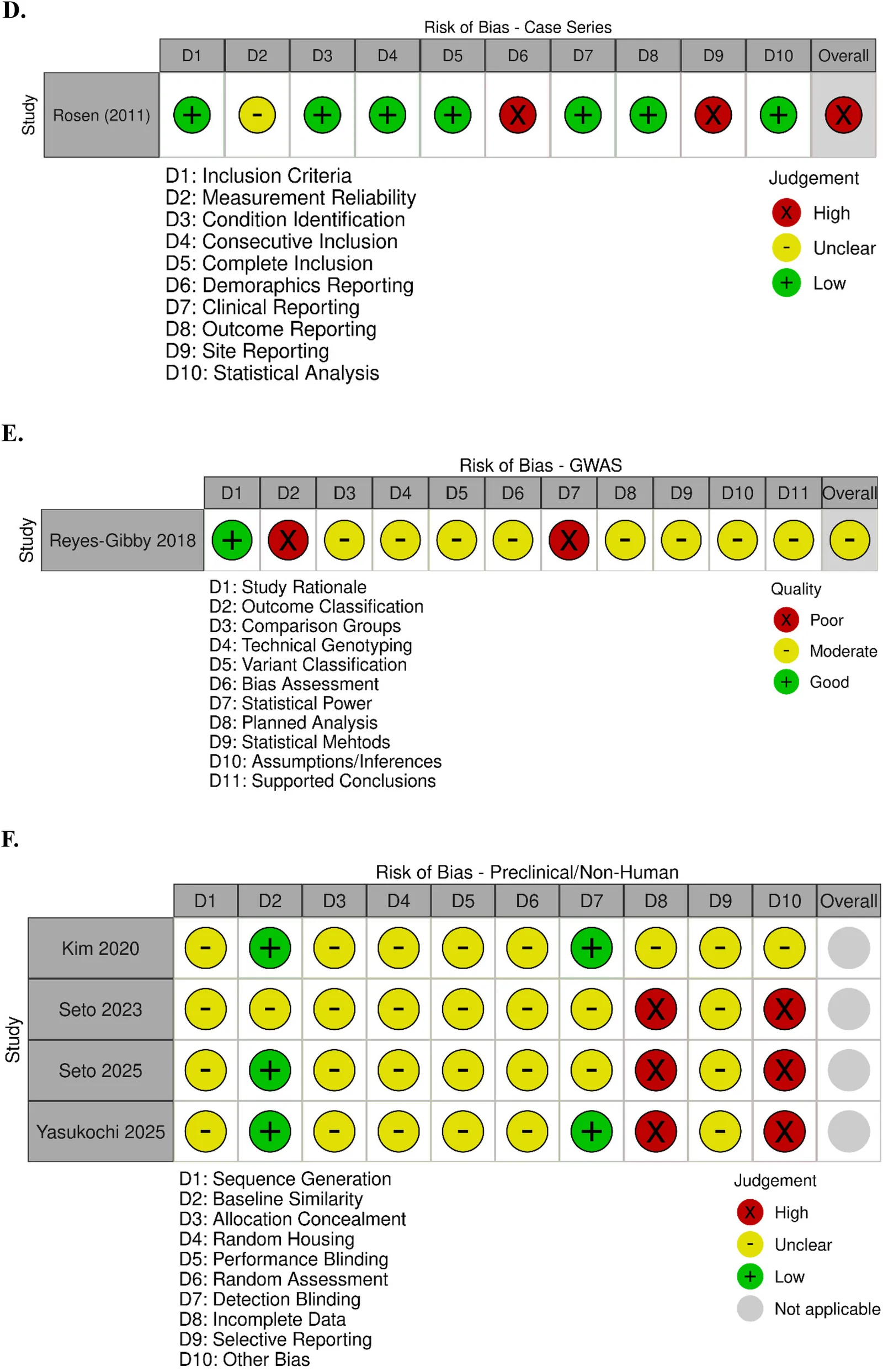
Risk of bias assessment of included studies in this systematic review. **(A)** JBI risk of bias assessment of cohort studies. **(B)** JBI risk of bias assessment of clinical trials studies. **(C)** JBI risk-of-bias assessment of cross-sectional studies. **(D)** JBI risk of bias assessment of case series study. **(E)** Q-Genie risk of bias assessment of genome wide association study (GWAS). **(F)** SYRCLE risk of bias of preclinical non-human experimental studies.

Among the clinical trials (*n* = 11), 8/11 (73%) were categorized as high risk-of-bias, 2/11 (18%) as low risk, and 1/11 (9%) as moderate risk (Figure 4B). The principal drivers of elevated risk in these trials were the participant blinding and provider blinding domains, each rated “No” in 7 of 11 trials (64%), and analytic limitations, with the intention-to-treat domain rated “No” in 6 of 11 trials (55%). Additionally, many trials did not clearly report whether follow-up was complete (Completeness of Follow-up domain: 6/11, 55% “Unclear”) or whether differences in follow-up between groups were appropriately analyzed.

The six analytical cross-sectional studies were appraised with the revised JBI checklist for that design (Figure 4C). Confounding was the dominant weakness: four of six studies (66.7%) did not identify potential confounders and five of six (83.3%) stated no strategy to address them. Only one study [Buffum et al. (88)] identified confounders in advance, adjusted for them across every outcome, and was rated adequate on all eight items. Statistical analysis was rated inadequate or unclear in four of six studies (66.7%), most often because of unadjusted multiple comparisons. Exposure and outcome measurement were comparatively strong: five of six studies (83.3%) measured the circadian exposure adequately, four using validated actigraphy protocols and three applying cosinor or comparable rhythmometric modelling. Overall appraisals were included for three studies, including with caution for one, seeking further information for one, and excluding from any synthesis of effect for one [Barni et al. (76)], in which neither the pain exposure nor the circadian outcome carried an operational definition.

The single included case series (1/1, 100%) was classified as high risk-of-bias due to incomplete reporting of participant demographic characteristics and lack of detailed information regarding the clinical setting in which cases were identified (Figure 4D). The qualitative study by Flynn et al. (34) met 7 of the 10 JBI critical appraisal criteria and was considered as moderate risk of bias. The main methodological limitations were the absence of a statement describing researcher positionality and insufficient consideration of the influence of the researchers on the research process, while participant representation, data analysis, ethical considerations, and interpretation of findings were adequately addressed.

The single genome-wide association study [Reyes-Gibby et al. (56)] was appraised with Q-Genie and scored 36 of a possible 77, placing it in the moderate quality band by one point above the poor-quality threshold of 35 (Figure 4E). The lowest-scoring items were classification of the outcome (2 of 7) and adequacy of statistical power (2 of 7). The neuropathy phenotype was defined solely by the presence of one of seventeen ICD-9 or ICD-10 codes, pooling diabetic, hereditary, postherpetic, infectious, ischemic optic and drug-induced neuropathy into a single binary case status, with no clinical examination, symptom instrument, or severity grade. The study reported 80% power to detect an odds ratio of 3.03, while three of its four reported associations had odds ratios between 0.51 and 1.59. Genotyping quality control, Hardy-Weinberg testing, sex concordance, cryptic relatedness, and ancestry verification were all described and reproducible, and these items carried the score.

Across all human studies, participant selection and outcome identification were generally well described; however, inadequate control of confounding in observational studies, incomplete reporting of follow-up, and limited implementation of blinding procedures in randomized trials represented the primary sources of systematic bias within the literature.

The four pre-clinical studies were appraised with the SYRCLE tool (Figure 4F). Six of the ten domains were rated unclear in all four studies (100%): sequence generation, allocation concealment, random housing, blinding of caregivers and investigators, random outcome assessment, and selective outcome reporting. In each case the judgement reflected absent reporting of the method rather than a documented methodological failure. Baseline characteristics were rated low risk in three studies (75%) and unclear in one (25%). Blinding of outcome assessors was rated low risk in two studies (50%) and unclear in two (50%); two studies explicitly reported blinding of the behavioral assessor, and one additionally reported blinded histological scoring. Incomplete outcome data was rated high risk in three studies (75%) and unclear in one (25%), reflecting unexplained variation in group sizes between outcomes, contradictions between methods text and figure legends, and no acknowledgement of attrition in any study. Other sources of bias were rated high risk in three studies (75%) and unclear in one (25%), driven by unit-of-analysis concerns, pooling or omission of comparator arms, and the use of analysis of variance across time points rather than a periodicity method to test for rhythmicity. No domain was rated low risk across all four studies, and no study reported a sample size calculation. Notably for a circadian review, cage placement was unreported in all four studies even though cage position determines light intensity and temperature, and only one study reported using dim red light for dark-phase handling.

## 4 Discussion

This systematic review examined current literature and evaluated clinical and preclinical evidence linking circadian biology with cancer- and cancer treatment-related pain, analgesic response, and neuropathy. Clinical findings were heterogeneous, with temporal variation in pain, analgesic requirements, and treatment-related neuropathy reported in several studies, while others found no significant associations. Chronotherapy studies suggested potential effects of treatment timing on neuropathy and neurotoxicity; however, most did not directly assess pain (59, 60, 62, 63, 79). Mechanistic evidence from human studies was predominantly hypothesis-generating, with limited direct mechanistic investigation of the proposed biological pathways. Importantly, only four preclinical studies directly investigated these relationships, highlighting a critical gap in the field. Overall, the findings suggest a potential but complex relationship between circadian biology and cancer-related pain that remains insufficiently characterized at a mechanistic level across the existing literature.

Our findings should also be considered in the context of recent reviews examining circadian biology and pain more broadly [Marelli et al. (67); Van Stallen et al. (68)]. These studies highlight increasing recognition of circadian influences on pain and analgesic response, while also demonstrating important gaps in the available evidence. Our systematic review extends this literature by focusing specifically on cancer- and cancer treatment-related pain, analgesic response, and pain-relevant neuropathy and by integrating clinical and preclinical evidence. Importantly, our findings demonstrate that direct mechanistic evidence in the oncology setting remains limited, with much of the human evidence being observational or hypothesis-generating. This highlights the need for studies specifically designed to evaluate circadian mechanisms and treatment timing in cancer-related pain.

The included studies spanned a wide range of designs and populations, with most focusing on mixed or advanced cancer cohorts. These populations are often characterized by uncontrolled pain and higher symptoms burden. Xia et al. reported that patients with advanced cancer often had low pain acceptance and experienced intense chronic pain accompanied by negative emotions and behaviors (102). Additionally, studies included in our review used a wide range of designs, most commonly cohort and other longitudinal observational studies, followed by clinical trials. Publications have increased over the past decade, indicating growing interest in circadian rhythms and cancer pain. However, most of the studies failed to examine or discuss the mechanisms behind this relationship.

Despite this limitation, several biological pathways were noted and hypothesized that may explain the relationship between circadian biology and cancer or cancer treatment-related pain or neuropathy. Evidence from both human and non-human studies suggests that circadian disruption is associated with increased inflammatory signaling and neuroinflammation as reported by Shin et al. and Kim et al. (97). Disturbed rhythmic regulation of immune responses may lead to continuous production of pro-inflammatory cytokines that can cause inflammatory pain and sensitize nociceptors and amplify pain perception (32, 37, 38). For example, IL-6 specifically demonstrated a positive correlation with pain intensity in patients with prostate cancer, linking systemic inflammation with cancer-related pain burden (40–42). Additionally, circadian disruption may further contribute to neuroinflammation, as clock genes regulate microglial activation and inflammatory signaling within the central nervous system (43, 44). Sustained neuroinflammatory states can enhance central sensitization and prolong pain responses. Experiments by Kim et al. demonstrated circadian oscillation in chemotherapy-induced peripheral neuropathy, mediated by lengthened DRG periods and increased TNF-α, IL-1 and IL-6 (97).

In addition, neuroendocrine dysregulation, particularly involving the HPA axis, was frequently proposed in our review as a key mechanism, with studies reporting blunted or altered cortisol rhythms in patients experiencing higher pain levels as reported by Ma et al. (1) and Chang et al. (90). Dysregulation of endocrine hormones such as cortisol and melatonin may impair endogenous pain-modulating systems and reduce the body's natural ability to counteract nociceptive signaling (98). There are changes in diurnal cortisol slopes and a blunted cortisol awakening response in patients with cancer (46, 47). These findings support the idea of HPA axis dysregulation, impaired cortisol rhythmicity, disruption of anti-inflammatory signaling, and reduction in stress modulation (48). All may increase pain intensity in patients with cancer.

Haak et al., illustrated the bidirectional relationship between sleep and chronic pain, demonstrating neurobiological evidence of mechanisms involved in pain modulation that include the immune system, melatonin changes, opioid system, HPA axis and nitric oxide signaling (103). Additionally, Hannibal et al. described how chronic stress persistently activates the HPA axis, leading to cortisol dysfunction, inflammation, and exacerbation of pain (104). Because cancer and its treatment are physiologically and psychologically stressful, a similar HPA and cortisol pathway may plausibly contribute to pain in this population, although this has not been directly tested. The results of the resent review in cancer pain align with literature findings in general pain across different diseases (1, 90, 93, 96).

Chronotherapy studies further suggest that the timing of anticancer treatment may influence neurotoxicity and neuropathic pain through circadian variation in drug metabolism, pharmacokinetics, and tissue susceptibility. Many processes involved in drug absorption, distribution, metabolism, and elimination exhibit circadian rhythmicity, potentially resulting in time-of-day-dependent differences in systemic and neuronal drug exposure (105, 106). This concept is supported by the preclinical studies included in our review. This concept is supported by the preclinical studies included in our review. Seto et al. (98) demonstrated that cisplatin-induced peripheral neuropathy varied according to dosing time, with differences in oxidative stress, mitochondrial dysfunction, and neuronal apoptosis, suggesting that circadian variation in tissue susceptibility to oxidative injury may contribute to treatment-related neuropathy. Similarly, dosing-time-dependent differences in paclitaxel-induced mechanical allodynia were associated with differences in plasma paclitaxel concentrations, supporting a potential role for circadian variation in drug metabolism and clearance (99). These mechanisms may interact with endogenous circadian regulation of neuronal and inflammatory pathways, thereby modifying susceptibility to chemotherapy-induced peripheral nerve injury and pain.

Emerging evidence also supports a potential role for clock genes in the modulation of cancer- and cancer treatment-related pain and neuropathy although direct clinical evidence remains limited. Circadian disruption of clock genes has been shown to dysregulate immune cell activity and cytokine release, particularly increasing mediators such as IL-6, TNF-α, and IL-1β, which are well-established drivers of pain signaling and peripheral/central sensitization (27, 38, 39). In the only human genetic association study identified in our review, Reyes-Gibby et al. identified *RORA* associated with neuropathy in HNC cancer (56). *RORA* is involved in circadian regulation as well as immune and inflammatory processes, suggesting a potential biological link between molecular clock function, neuroinflammation, and susceptibility to treatment-related neuropathy (107). Complementing this clinical association, Kim et al. demonstrated intrinsic circadian oscillations in the dorsal root ganglion involving core clock components, including *PER2* and *BMAL1*, and showed that paclitaxel altered DRG circadian rhythmicity and the time-dependent expression of clock- and pain-related genes (97). Additional preclinical evidence supports a role for *PER2* in pain regulation beyond the cancer setting. Yamakawa et al. demonstrated a role for *PER2* in neuropathic pain through regulation of the endocannabinoid system (26), while Bigliardi et al., identified interactions between opioid receptor signaling and *PER2* regulation in an *in vitro* keratinocyte model (108). These findings suggest that clock genes may influence pain through interconnected neuronal, neuroimmune, inflammatory, endocannabinoid, and opioid-signaling pathways.

These findings suggest several potential clinical implications of circadian biology in cancer pain management. Evidence from chronotherapy studies suggests that treatment timing may influence treatment-related neuropathic pain and neurotoxicity, raising the possibility that optimizing the timing of anticancer therapy could reduce toxicity while maintaining treatment efficacy (59–61, 63). Observational studies also suggested that circadian characteristics could potentially contribute to risk stratification and identification of patients who may require closer symptom monitoring or earlier supportive interventions (109, 110). Additionally, several studies reported diurnal variation in opioid and analgesic requirements, suggesting that time-of-day may influence pain perception and pharmacologic response (76, 78, 82). Together, these observations support the potential for incorporating circadian biology into personalized pain management strategies and treatment planning in oncology.

The overall methodological quality of the evidence was limited, with important sources of high or unclear risk of bias across clinical and preclinical studies. Human studies were primarily limited by inadequate control of confounding, incomplete follow-up, missing-data concerns, and limited blinding, while the single genome-wide association study was of moderate quality (56). Preclinical studies were characterized by insufficient reporting of randomization, allocation concealment, housing, and blinding, resulting predominantly in unclear risk-of-bias judgments rather than documented methodological failures (97–100). More substantive concerns included incomplete outcome data, potential unit-of-analysis issues, and limitations in statistical approaches for assessing rhythmicity. Mechanistic pathways were also frequently inferred rather than directly tested. These limitations highlight the need for more rigorous, standardized, and mechanistically driven studies.

### 4.1 Future directions

Future research should integrate objective circadian biomarkers, including melatonin, cortisol, activity-rest rhythms, and clock-gene expression, with standardized assessment of cancer- and treatment-related pain and neuropathy. Circadian measurements should follow established methodological guidance and account for behavioral and environmental influences (111). Mechanistic studies are needed to clarify how circadian regulation influences neuronal, neuroimmune, endocrine, and treatment-related pathways underlying these outcomes. Standardized circadian and pain assessments and multi-omics approaches may facilitate identification of predictive biomarkers and improve comparability across studies. Painful neuropathy should also be distinguished from neuropathy or neurotoxicity without reported pain. Finally, adequately powered prospective trials are needed to determine whether circadian-informed treatment or analgesic timing can reduce pain, neuropathy, and treatment-related toxicity (112).

### 4.2 Limitations

Several limitations should be considered when interpreting the findings. First, the overall quality of the included studies was limited across both human and preclinical studies. Human studies were particularly limited by inadequate control of confounding, limited blinding, incomplete follow-up, and missing-data concerns, reducing the certainty and generalizability of the clinical findings. Second, the heterogeneity across studies in cancer type, population, and study design limited comparability and precluded meta-analysis (113). Third, pain and neuropathy were assessed inconsistently and were used differently across studies, either as exposures, associated factors, or outcomes. Additionally, the inclusion of both direct pain outcomes and indirect pain-related outcomes, such as neuropathy and neurotoxicity, broadened the evidence base but further increased heterogeneity. The literature search was restricted to English-language publications and primarily to PubMed and Web of Science; therefore, relevant studies published in other languages or indexed exclusively in other databases may have been missed. Fourth, although preclinical studies provided valuable mechanistic insights, their direct translation to clinical settings is limited due to differences in experimental conditions and lack of standardized models. Lastly, mechanistic evidence was limited, and findings from preclinical models may not directly translate to patients with cancer.

## 5 Conclusion

The available evidence on circadian rhythm and cancer-related pain suggests a possible association between circadian biology and cancer- or cancer treatment-related pain, analgesic response, and neuropathy, through interacting neuronal, neuroimmune, inflammatory, endocrine, metabolic, and molecular mechanisms. heterogenous findings, methodological limitations, high risk-of-bias, and limited direct mechanistic evidence preclude firm causal or clinical conclusions. Further prospective and mechanistic studies are needed to determine whether circadian-informed approaches can improve cancer pain management and patient quality of life.

## Data Availability

The original contributions presented in the study are included in the article/Supplementary Material, further inquiries can be directed to the corresponding author.
